# Optimizing Kernel Extreme Learning Machine based on a Enhanced Adaptive Whale Optimization Algorithm for classification task

**DOI:** 10.1371/journal.pone.0309741

**Published:** 2025-01-03

**Authors:** ZeSheng Lin

**Affiliations:** Vocational Training Center, FoShan Open University, FoShan, Guangdong Province, China; University of Mosul, IRAQ

## Abstract

Data classification is an important research direction in machine learning. In order to effectively handle extensive datasets, researchers have introduced diverse classification algorithms. Notably, Kernel Extreme Learning Machine (KELM), as a fast and effective classification method, has received widespread attention. However, traditional KELM algorithms have some problems when dealing with large-scale data, such as the need to adjust hyperparameters, poor interpretability, and low classification accuracy. To address these problems, this paper proposes an Enhanced Adaptive Whale Optimization Algorithm to optimize Kernel Extreme Learning Machine (EAWOA-KELM). Various methods were used to improve WOA. As a first step, a novel adaptive perturbation technique employing T-distribution is proposed to perturb the optimal position and avoid being trapped in a local maximum. Secondly, the WOA’s position update formula was modified by incorporating inertia weight *ω* and enhancing convergence factor *α*, thus improving its capability for local search. Furthermore, inspired by the grey wolf optimization algorithm, use 3 excellent particle surround strategies instead of the original random selecting particles. Finally, a novel Levy flight was implemented to promote the diversity of whale distribution. Results from experiments confirm that the enhanced WOA algorithm outperforms the standard WOA algorithm in terms of both fitness value and convergence speed. EAWOA demonstrates superior optimization accuracy compared to WOA across 21 test functions, with a notable edge on certain functions. The application of the upgraded WOA algorithm in KELM significantly improves the accuracy and efficiency of data classification by optimizing hyperparameters. This paper selects 7 datasets for classification experiments. Compared with the KELM optimized by WOA, the EAWOA optimized KELM in this paper has a significant improvement in performance, with a 5%-6% lead on some datasets, indicating the effectiveness of EAWOA-KELM in classification tasks.

## 1 Introduction

Data classification is a core task in machine learning domain [1]. Extreme learning machines (ELM) [2], as a prompt and efficient machine learning algorithm, have been widely used in classification and prediction tasks in various fields [3]. By merging kernel functions with the ELM method, the KELM approach provides significant enhancements. With their robust generalization performance and rigorous mathematical basis, Kernel based learning methods can effectively boost the adaptability and accuracy of the model, without sacrificing the benefits of ELM [4]. Nevertheless, The KELM model may encounter local minimum [5] problems and exhibit weak controllability because its generalization performance is closely related to parameter selection [6–8], so metaheuristic algorithms need to be used to improve model prediction performance. Metaheuristic optimization methods such as particle swarm optimization(PSO) [9], grey wolf optimization algorithm(GWO) [10], and firefly algorithm(FA) [11] are frequently employed in fine-tuning the parameters of KELM. The pursuit of the global optimum poses a difficulty for all metaheuristic algorithms as they strive to maintain a balance between exploration and exploitation [12]. Thus, they may require more iterations and methods to discover the best worldwide solution. The search for the optimal algorithm for a given optimization problem continues to be a challenge. A swarm intelligence optimization technique called the whale optimization algorithm (WOA) was suggested in 2016 [13]. It optimizes and solves the objective function through the simulation of how whale groups search for food in the ocean. Compared with many heuristic optimization algorithms, the successful global optimization performance of WOA can be attributed to its adoption of natural and biological behaviors seen in whales. Therefore, the whale optimization algorithm has been a popular choice among researchers for enhancing the performance of KELM in numerous studies [14–16]. Although these studies strong advantages to solve many problems, they also come with limitations like slow convergence and a tendency to reach local optimal solutions. It still has certain flaws when solving complex engineering problems. As an illustration, it can be simple to get stuck in local optima, resulting in fluctuations in the performance of the model’s predictions. Therefore, the algorithm needs to be further improved to improve the stability of the optimization function.

To mitigate these problems, this paper presents a technique for optimizing the performance of KELM in classification task based on an enhanced adaptive whale optimization algorithm (EAWOA-KELM). An enhanced version of the WOA algorithm was proposed through the implementation of various strategies such as T-distribution probability perturbation, Levy flight, novel nonlinear control parameters, and average position updating. In this study, 21 test functions from three benchmark functions sets were used to assess the effectiveness of EAWOA. Finally, the proposed model is ultimately applied to categorize data on a range of social issues, serving as strong evidence for its reliability and superiority.

The arrangement of this paper is as stated in the following: Relevant studies are discussed in Section 2, while Section 3 presents an overview of the structure and working mechanisms of KELM and WOA. The proposed EAWOA and its improvement details are introduced in Section 4. Section 5 involved a series of experiments and discussions aimed at confirming the efficacy of EAWOA and its enhancement of KELM. In conclusion, Section 6 summarizes the main findings and future plans for the entire study.

## 2 Related work

Nowadays, machine learning algorithms like ELM are extensively utilized for prediction and classification purposes, and the way their parameters are set significantly influences the algorithm’s results. Hence, various studies employ intelligent optimization algorithms like WOA algorithm to identify the most suitable parameters for machine learning algorithms.

Intelligent statistical technologies represented by Extreme Learning Machines(ELM) have gained widespread usage in the domains of forecasting [17] and categorization [18]. ELM is a feedforward neural network with a single hidden layer, but it was later developed to incorporate deep structures which can perform representation learning by the stacking encoder [19]. Unlike other neural networks, ELM stands out for its speed as it has fewer layers and does not utilize backpropagation in parameter adjustment. Over the past few years, ELM has been a prominent subject of investigation due to its remarkable efficiency and user-friendly nature. It has also been effectively utilized in numerous research domains, including missing data handling, imbalance correction, and activity recognition [20]. The development of KELM is rooted in ELM and involves the conversion of ELM’s random mapping to a kernel-based mapping, which not only addresses the issue of requirement to decide the neurons number of the hidden layer, but also yields strong generalization capabilities [21]. Although great progress has been made on both theory and practice of ELM, one issue that is still not well considered is how to select an optimal kernel parameters when kernel tricks are applied to ELM. In other words, choosing appropriate parameters is very important for KELM performance. Among them, using metaheuristic algorithms to select parameters is a current mainstream method [22].

The process of optimization is centered around identifying the most ideal solution or parameter value from a group of choices under certain condition. Metaheuristic algorithms usually draw on the wisdom of natural sciences such as biology or physics to find optimal solutions [23]. Nevertheless, there are restrictions regarding the rapidity and consistency of algorithm convergence. Researchers continue to propose metaheuristic algorithms with higher efficiency. Based on various sources of inspiration, metaheuristic algorithms can typically be grouped into three categories: evolution, physics, and group dynamics. Algorithms based on biological evolution include genetic algorithm (GA) [24], differential evolution (DE) [25], evolutionary strategy(ES) [26], etc. Algorithms based on physical principles include water evaporation optimization(WEO) [27], simulated annealing (SA) [28], charged system search (CSS) [29], etc. Typical representatives of swarm intelligence optimization algorithms include PSO, GWO, FA, and others. As a result of observing the survival tactics of animals, numerous innovative optimization algorithms have been produced, including Sparrow Search Algorithm(SSA) [30], Gorilla Troops Optimizer(GTO) [31], WOA, etc. Moreover, various metaheuristic algorithms or concepts have distinct benefits and can work well together. Accordingly, a large number of researchers have suggested various improved hybrid algorithms that combine ideas from different algorithms or natural phenomena to enhance the effectiveness of the original algorithms, such as Improved Corrective Smoothed Particle Method [32], Exploratory Cuckoo Search [33], and Improved Sparrow Search Algorithm with HDPM [34].

With its proven success in solving optimization problems, the WOA algorithm has gained widespread recognition in fields like traffic network [35], path planning [36, 37]. In spite of its evident effectiveness over other advanced algorithms, it remains challenged by factors such as sluggish convergence, inadequate exploration potential, and being trapped in local optimal solutions [38]. For example, WOA uses a coefficient vector |*A*| that is heavily influenced by the convergence factor *α*, which determines whether the WOA algorithm performs exploration phase or encircling prey. In WOA, the exploration phase is chosen when. But as the number of iterations increases to the latter half, the WOA algorithm tends to only execute the development phase and weakens the exploration process [38]. Many strategies have been put forth to elevate the efficiency of WOA. Chen et al. proposed several multistrategy approaches to better balance the exploration phase or encircling prey [39, 40]. some researcher use Levy flight trajectory to the WOA algorithm to to increase diversity in solution [41, 42]. Fan et al. introduced a novel ESSAWOA algorithm which incorporates enhanced SSA and WOA [43]. By combining WOA and PSO, Huang et al. proposed whale particle optimization (WPO) to promote a greater range of particles [44]. Furthermore, there are advanced whale optimization methods that take cues from physical phenomenon. For instance, Tang et al. proposed a novel WOA algorithm that incorporates the concept of atom-like differential evolution, which defines whale behavior as quantum mechanical behavior [45]. Utilizing the benefits of alternative algorithms can help overcome the limitations of WOA. Throughout the development of WOA, Bilal H et al. developed a refined variation of WOA called Island-based Whale Optimization Algorithm by integrating island models to boost diversity [46]. Subsequently, they combined the hill-climbing algorithm with the WOA algorithm, resulting in the emergence of WOABHC. The hill-climbing algorithm serves to uphold the diversity within the algorithm [47].

Although the above algorithms have made significant progress in improving WOA, there is still potential development for WOA in global optimization, convergence speed, and other aspects.

## 3 Methods

**Fig 1:**
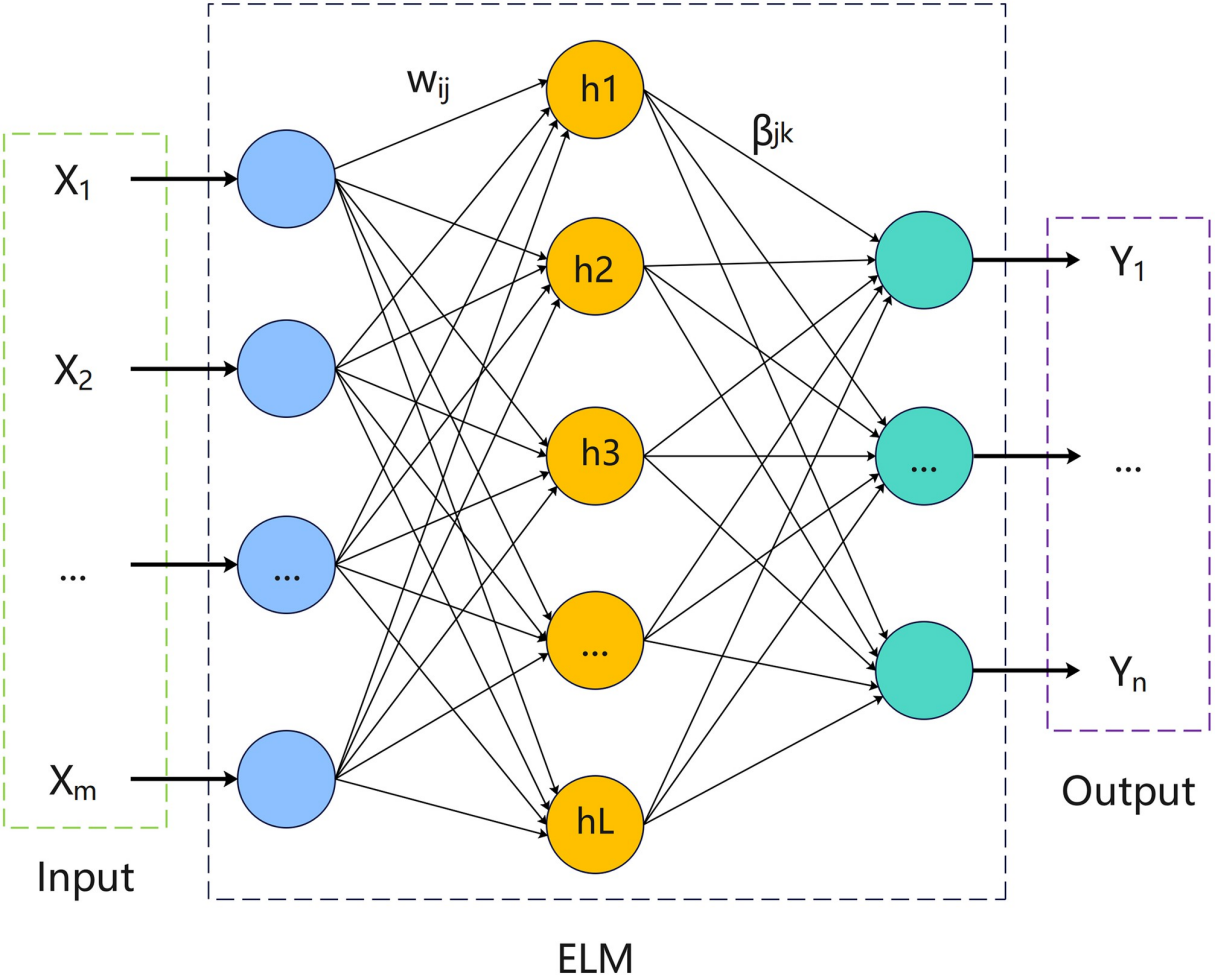
Model of ELM.

This section presents the fundamental principles behind the models and algorithms discussed in this paper. The details are outlined as follows: Section 3.1 provides a thorough examination of the architecture of KELM. Section 3.2 provides a detailed introduction to the whale optimization algorithm, offering an in-depth exploration of its three main stages: encircling prey, exploitation phase and exploration phase. Fig 1 displays the schematic diagram of KELM, while Algorithm 1 shows the schematic diagram of WOA. By delving into these components, this paper seeks to shed light on the inventive approach of blending KELM and WOA to deal with parameter optimization challenges and enhance predictive accuracy.

### 3.1 Kernel Extreme Learning Machine

ELM is a highly efficient neural network known for its exceptional learning capabilities and performance. Due to its single hidden layer network and absence of backpropagation algorithm, ELM is able to achieve impressive results. Fig 1 illustrates the network design of ELM, which is comparable to that of a neural network in many ways. Except that neural networks use gradient descent to adjust parameters, while ELM’s parameters are manually set, so its running speed is much faster. However, it is prone to problems such as unstable training results and unsatisfactory generalization ability. In order to fortify the system and enhance its overall effectiveness, Kernel Extreme Learning Machine (KELM) adopts kernel mapping in place of the random mapping used in Extreme Learning Machine (ELM), leading to improved performance and generalization capability.

The mathematical representation of ELM can be simplified to Eq (1).

$$\begin{array}{c} f(x)=h(x)\beta =H\beta \end{array}$$ (1)

where x and f(x) represent the input and output of the model respectively, h(x) or H represents the output matrix obtained by inputting x into the hidden layer, *β* defines the weight matrix connecting the hidden neurons and output layer which can be expressed as Eq (2):

$$\begin{array}{c} \beta ={\left(HH^{T}+\frac{I}{C}\right)}^{-1}H^{T}E \end{array}$$ (2)

where *H*^*T*^ is the transposed matrix of H, I is the identity matrix, C is the regularization coefficient, and E is the expected output matrix. The kernel function *Ω* in KELM can be expressed as Eq (3):

$$\begin{array}{c} \Omega =HH^{T}=h\left(x_{i}\right)h\left(x_{j}\right)=k\left(x_{i},x_{j}\right) \end{array}$$ (3)

This paper uses the Radial Basis Function(RBF) as the kernel function, and the calculation formula is:

$$\begin{array}{c} \Omega =k\left(x_{i},x_{j}\right)=exp\left(-\frac{{∥x_{i}-x_{j}∥}^{2}}{s}\right) \end{array}$$ (4)

where *S* = 2*δ*^2^, the standard output of KELM can be expressed as Eq (5)):

$$\begin{array}{c} F\left(x\right)=H\beta =\left[\begin{array}{c} k\left(x,x_{1}\right)\\ \ldots \\ k\left(x,x_{M}\right) \end{array}\right]{\left(\frac{I}{C}+\Omega \right)}^{-1}E \end{array}$$ (5)

In fact, the effectiveness of the KELM model relies heavily on the chosen values for the regularization coefficient C and kernel function S. In other words, different (C, S) combinations will cause a direct influence on the KELM’s forecasting capability. Therefore, choosing the appropriate (C, S) combination is very important for KELM. The KELM model poses a nonlinear problem, making it a daunting task to obtain the best solution using conventional methods. To deal with this problem effectively, the paper puts forth a innovative WOA to effectively optimize the KELM parameters.

### 3.2 Whale Optimization Algorithm (WOA)

WOA is a biologically-inspired algorithm created for optimization purposes. By mimicking the social habits of whale groups, it is able to effectively search globally and optimize locally for complex optimization problems. The following are the main principles of the WOA:

Suppose there is a group of whales searching in a d-dimensional space, then the whale numbered i can be expressed as $X_{i}=\left(x_{i}^{1},x_{i}^{2},\ldots x_{i}^{d}\right)$. Each whale’s position serves as a solution to the problem. The entire process of the WOA can be divided into 3 different stages: encircling prey, exploitation phase, search for prey.

#### 3.2.1 Encircling prey

This stage simulates the behavior of whales contracting and surrounding their prey after discovering it. Nevertheless, due to the unknown optimal location in the search space, WOA algorithm presumes the prey’s position in the current iteration is the optimal position which obtains minimum fitness value. Use to represent this prey and other whales will surround the target prey. The calculation formula for this process is as Eqs (6) to (9):

$$\begin{array}{c} D=|C\cdot X^{*}\left(t\right)-X\left(t\right)| \end{array}$$ (6)

$$\begin{array}{c} X\left(t+1\right)=X^{*}\left(t\right)-A\cdot D \end{array}$$ (7)

$$\begin{array}{c} A=2a\cdot r_{1}-a \end{array}$$ (8)

$$\begin{array}{c} C=2r_{2} \end{array}$$ (9)

where t is the current iteration number, A and C are coefficients, the whale’s current position is *X*(*t*), while its updated position is *X*(*t* + 1). X* is the whale position that obtains the optimal value. *α* is the convergence factor, its expression is $\alpha =2-2\frac{t}{t_{max}}$, where *t*_*max*_ represents the iterations number. *α* linearly decreases from 2 to 0 during the iteration process. *r*_1_ and *r*_2_ are random vectors from 0 to 1.

#### 3.2.2 Exploitation phase

The exploitation phase demonstrates another way for whales to approach prey, which is complementary to the way they surround prey. The behavior involves spiraling motion, commonly referred to as spiral bubble net feeding. The logarithmic spiral model can be defined as Eqs (10) and (11):

$$\begin{array}{c} X\left(t+1\right)=D\cdot e^{bl}\cdot cos\left(2\pi l\right)+X^{*}\left(t\right) \end{array}$$ (10)

$$\begin{array}{c} D=X^{*}\left(t\right)-X\left(t\right) \end{array}$$ (11)

#### 3.2.3 Search for prey (exploration phase)

The utilization of the first two bracketing strategies limits the thorough examination of the space, resulting in WOA getting trapped in local optimality. To combat this limitation, WOA introduces a random strategy in exploration phase. When A is greater than 1, a whale will be chosen at random as prey, and other whales will come closer to it. This technique will broaden the whale’s search area and lead to improved locations. This process is expressed by Eqs (12) and (13). It is similar to the process of Eqs (6)–(9), except that the prey surrounded is different.

$$\begin{array}{c} D=C\cdot X_{rand}-X \end{array}$$ (12)

$$\begin{array}{c} X\left(t+1\right)=X_{rand}-A\cdot D \end{array}$$ (13)

The pseudo code of the WOA algorithm is presented in Algorithm 1.

**Algorithm 1** Whale Optimization Algorithm

1: Initialize the position of the whale group: *X*_*i*_(*i* = 1, 2, …*k*)

2: Calculate the fitness value of each whale, and use the whale with the smallest fitness value as the optimal whale X*

3: **while** *t* < *T*_*max*_ **do**

4:  **for** each whale **do**

5:   Update parameters *α*, *A*, *C*, *l*, and *p*

6:   **if** *p* < 0.5 **then**

7:    **if** |*A*| < 1 **then**

8:     Employ Eq (7) to update the current position of the whale

9:    **else**

10:     Select a random whale *X*_*rand*_

11:     Employ Eq (13) to update the current position of the whale

12:    **end if**

13:   **else**

14:    Employ Eq (10) to update the current position of the whale

15:   **end if**

16:  **end for**

17:  Apply boundary constraints to each search agent and Calculate the fitness of each whale

18:  Update the optimal whale X*

19:  *t* ← *t* + 1

20:  **end while**

21: **return** optimal whale X*

## 4 Enhanced Adaptive Whale Optimization Algorithm

**Fig 2:**
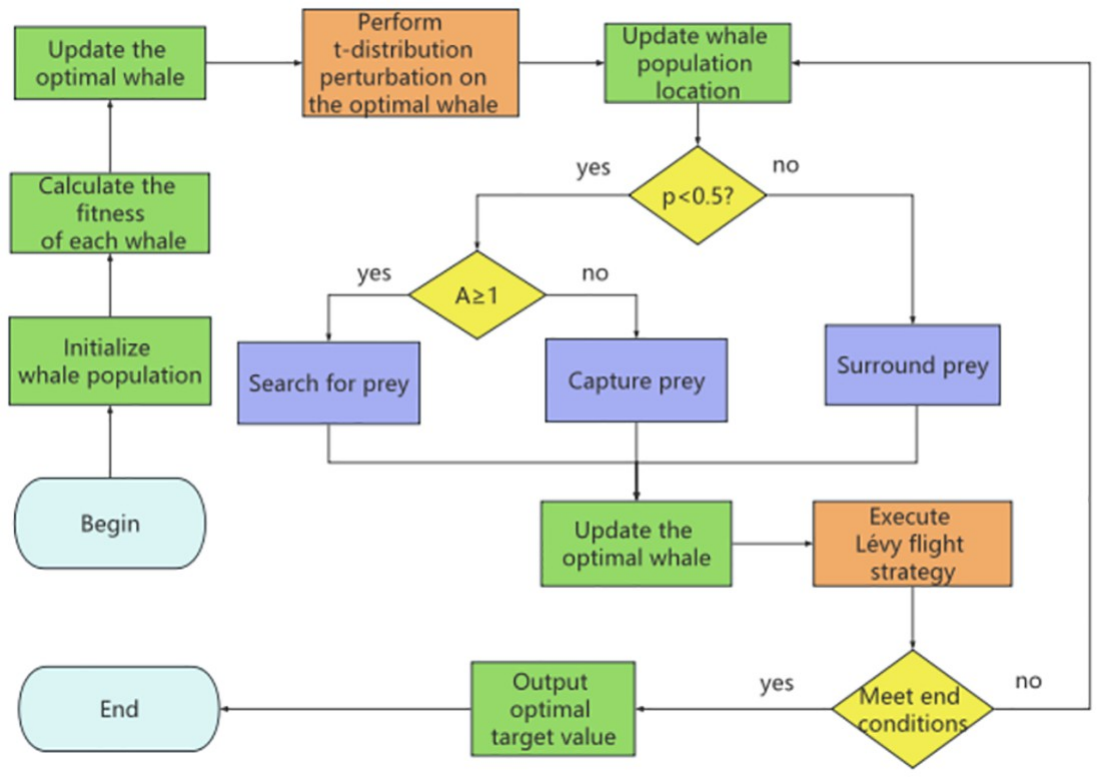
The flowchart of the proposed EAWOA.

The overall flowchart of the proposed EAWOA is shown in Fig 2. In WOA’s encircling prey phase and exploitation phase, other whales naturally move towards the optimal whale. By mutating the optimal whale, the algorithm’s performance can be significantly improved. Hence, a novel perturbation strategy based on adaptive T-distributions has been developed to perturb the optimal position to generate better solutions. Secondly, the convergence factor *α* is changed from linear decrease to nonlinear decrease, and a similar inertia weight *ω* is added. With this improvement, the algorithm is now able to conduct flexible searches at various stages. In addition, inspired by the grey wolf optimization algorithm, this paper uses the average position of three outstanding whales to replace the original random whale in the prey search stage. This process is similar to having three excellent hunters of whales surround their prey, enhancing the probability of the WOA algorithm finding the better value in the decision space. Finally, a innovative Levy flight was performed on the whale positions to improve global searching ability. This new Levy flight plan allows whale optimization algorithms to explore better solutions in different directions, enhancing the diversity of whale populations.

### 4.1 Adaptive T-distribution perturbation strategy

#### 4.1.1 T-distribution perturbation strategy

T-distribution is an important type of distribution in statistics which commonly utilized in statistical inference for small sample sizes, parameter estimation and hypothesis testing. The T-distribution’s probability density function can be described by Eq 14.

$$\begin{array}{c} f\left(t\right)=\frac{\Gamma (\frac{\nu +1}{2})}{\sqrt{\nu \pi }\;\Gamma (\frac{\nu }{2})}{\left(1+\frac{t^{2}}{\nu }\right)}^{-\frac{\nu +1}{2}} \end{array}$$ (14)

where *v* is the number of degrees of freedom and Γ is the gamma function.

#### 4.1.2 Adaptive T-distribution perturbation strategy

In this paper, we proposed a novel T-distribution perturbation to strengthen the optimal whale individual’s spatial exploration capability. With the potential for falling into local optimal dilemma in the later stages of iteration, it is advisable to improve the perturbation strategy during this time. The process of the adaptive t distribution strategy is shown as Eqs (15) and (16). where *C*1 = 0.64, *C*2 = 0.04 in this paper. *trnd*(*t*) is the T-distributed random number whose degree of freedom parameter is the number of iterations.*trnd*(*t*) represents a random vector generated from T-distributed, and its degree of freedom is the current number of iterations. A coefficient *s* between (0, 1) is implemented to moderate the disturbance intensity of the optimal whale. As the iterations increases, *s* becomes smaller, thus (1 − *s*) becomes larger, indicating that the degree of variation in the later period increases, which can better help the optimal whale position escape the local optimum in the later rounds.

$$\begin{array}{c} s=\sqrt{c1}-\sqrt{c2}\cdot {\left(\frac{t}{T_{MAX}}\right)}^{2} \end{array}$$ (15)

$$\begin{array}{c} X_{new}=X_{best}\cdot \left[s+\left(1-s\right)\times trnd\left(t\right)\right] \end{array}$$ (16)

In order to guarantee an improved position after the disturbance, a greedy approach is implemented after the disturbance. The fitness value of the new and old positions is compared to determine if a position update is necessary. Eq (17) represents the greedy strategy:

$$\begin{array}{c} X_{best}=\{\begin{array}{cc} X_{new}, & f\left(X_{new}\right)\leq f\left(X_{best}\right)\\ X_{best}, & f\left(X_{new}\right)>f\left(X_{best}\right) \end{array} \end{array}$$ (17)

### 4.2 Dynamic adaptive weight adjustment

#### 4.2.1 Nonlinear control parameter

In general, the swarm intelligence optimization algorithm is comprised of two separate stages: global search and local search. Ideally, algorithms use strong local search capabilities to determine the optimal value’s spatial range within the decision space, and then use local search to obtain the precise value. Therefore, the key to the algorithm achieving high search performance is how to effectively coordinate exploration and development capabilities. However, the original WOA algorithm’s control parameter *α* is linearly reduced, limiting its ability to fully utilize search capabilities. Hence, this paper proposes a nonlinear control parameter *α*. It is expressed as Eqs (18) and (19).

$$\begin{array}{c} \beta_{\left(t\right)}=e^{\beta_{min}}+cos{\left(\frac{\pi }{2}\cdot \frac{t}{t_{max}}\right)}^{2.5}\left(e^{\beta_{max}}-e^{\beta_{min}}\right) \end{array}$$ (18)

$$\begin{array}{c} \alpha =\frac{{\left(ln\beta_{\left(t\right)}\right)}^{c}}{b} \end{array}$$ (19)

where *β*_*max*_ = 2.0, *β*_*min*_ = 0, *b* = 2, *c* = 0.

**Fig 3:**
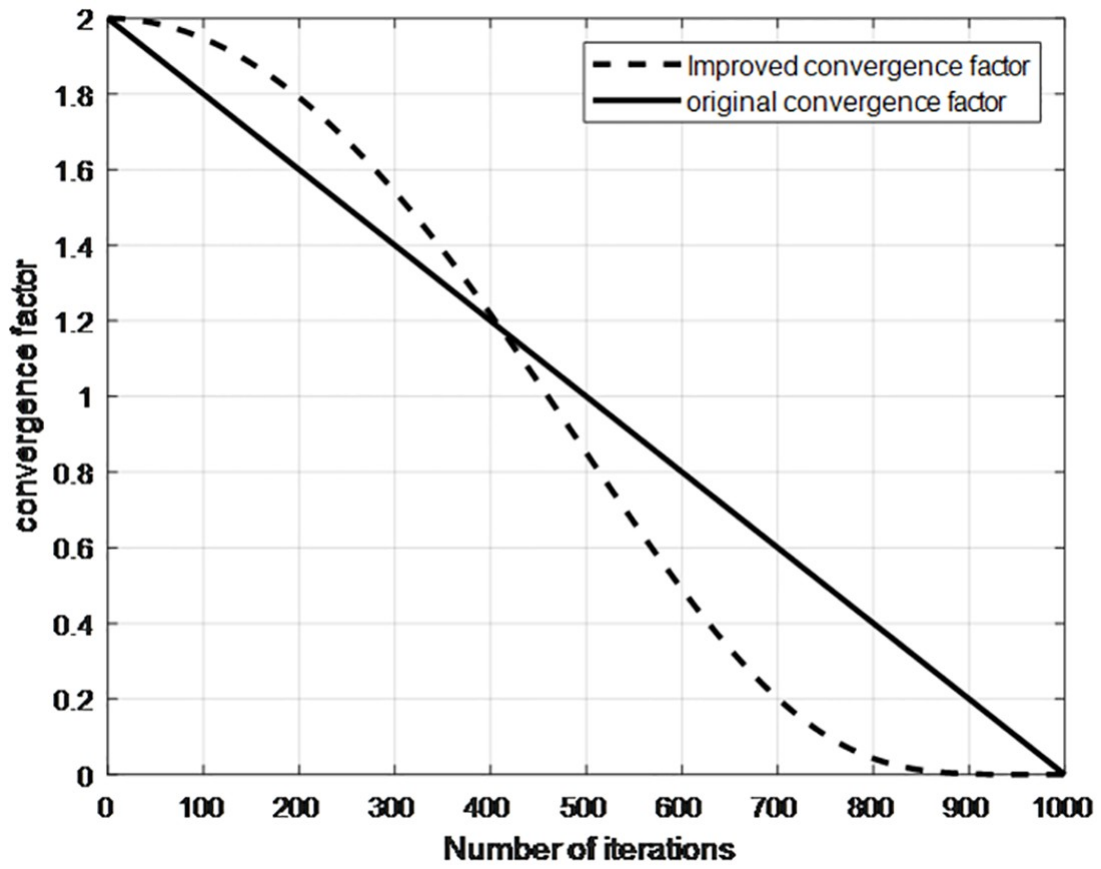
Convergence factor *α* change graph.

Fig 3 illustrates the changes in the convergence factor *α* both before and after improvement. This change of *α* enables the whale swarm to sustain bigger movements during the initial phases and smaller adjustments during the later part. It can be seen from Fig 3 the control parameter *α* changes nonlinear and dynamically. In contrast to the initial control parameter *α*, the enhanced control parameter *α* is initially larger and gradually becomes smaller, which enables the algorithm to perform extensive global and local searches during these two phases respectively.

#### 4.2.2 The adjustment weight *ω*

To achieve a more harmonious balance between exploitation and exploration stages, the adjustment weight *ω* is set as formulas 20 and 21, where *β*_*max*_ = 0.9, *β*_*min*_ = 0.4.

$$\begin{array}{c} \beta_{t}=e^{\beta_{min}}+cos{\left(\frac{\pi }{2}\cdot \frac{t}{t_{max}}\right)}^{2.5}\left(e^{\beta_{max}}-e^{\beta_{min}}\right) \end{array}$$ (20)

$$\begin{array}{c} \omega_{\left(t\right)}=ln\beta_{\left(t\right)} \end{array}$$ (21)

Similar to the principle of the nonlinear control parameters *α*, the adaptive weight adjustment *ω* further heightens algorithm’s effectiveness in global and local search. Apply *ω* to the three position update Eqs 7, 10 and 13, and get the following position update Eqs 22–24. As the number of iterations increases, *ω* decreases nonlinearly from 0.9 to 0.4, which helps each individual to make larger updates in the later stage and thus escape from the local optimum.

$$\begin{array}{c} X\left(t+1\right)=\omega \cdot X^{*}\left(t\right)-A\cdot D \end{array}$$ (22)

$$\begin{array}{c} X\left(t+1\right)=D\cdot e^{bl}\cdot cos\left(2\pi l\right)+\omega \cdot X^{*}\left(t\right) \end{array}$$ (23)

$$\begin{array}{c} X\left(t+1\right)=\omega \cdot X_{rand}-A\cdot D \end{array}$$ (24)

### 4.3 Learning strategy based on Levy random flight

The Levy flight model is an efficient form of random walk in enhancing algorithm diversity due to its random direction and length of motion. Many creatures in nature adopt this random walk strategy to increase the possibility of finding food. Inspired by this, Levy flight can be introduced into whale movements to increase the search possibility of the algorithm. This paper introduces a learning strategy to perform Levy flight on the particles after the updated position. Its expression is as Eqs 25 and 26, where *A*_1_ and *C*_1_ have the same meaning as *A* and *C* in encircling prey phase. *step* represents the vector of Levy flight, *β* = 1.5, and *μ* and *v* represent random vectors with the same dimensions as whale individuals.

$$\begin{array}{c} X_{new}^{i}=X^{i}+A_{i}*step⊕\left(X^{i}-C_{1}*X_{best}\right) \end{array}$$ (25)

$$\begin{array}{c} step=\frac{\mu }{{\left|v\right|}^{\frac{1}{\beta }}} \end{array}$$ (26)

### 4.4 New random whale in exploration phase

Many algorithms will have a strategy to reduce local optimal situations. And this strategy in WOA is expressed as Eq 24. That is the choosing of a random whales instead of the optimal whale. Despite this, if the chosen whale falls into a poor space, it will cause a gathering of other poor-performing whales. And the already converged algorithm will return to a scattered state, resulting in a waste of computing resources. Inspired by the grey wolf optimization algorithm, this paper selects the 2nd to 4th best individual whales as hunters, uses their average value as prey and replaces the original random whales with it. It can preserve the randomness of the algorithm while allowing the algorithm to search towards a more ideal space. This process is expressed by Eq 27, where *X*_*rank*2_, *X*_*rank*3_ and *X*_*rank*4_ respectively represent the whale positions with fitness values ranking 2nd to 4th.

$$\begin{array}{c} X_{rand}=\frac{X_{rank2}+X_{rank3}+X_{rank4}}{3} \end{array}$$ (27)

By enhancing the methods described above, this paper has successfully developed the EAWOA model. Algorithm 2 contains the pseudo code for the EAWOA algorithm.

**Algorithm 2** Enhanced Adaptive Whale Optimization Algorithm (EAWOA)

1: Initialize the position of the whale group: *X*_*i*_(*i* = 1, 2, …*k*)

2: Calculate the fitness value of the whale, and use the whale with the smallest fitness value as the optimal whale X*

3: **while** *t* < *T*_*max*_ **do**

4:  Perform adaptive T-distribution perturbation on the optimal whale (Eq 16)

5:  **for** each whale **do**

6:   Update parameters *α* (Eq 19), *w* (Eq 21), *A*, *C*, *l*, and *p*

7:   **if** *p* < 0.5 **then**

8:    **if** |*A*| < 1 **then**

9:     Employ Eq 22 to update the current position of the whale

10:    **else**

11:     Employ Eq 27 to obtain the random whale

12:     Employ Eq 24 to update the current position of the whale

13:    **end if**

14:   **else**

15:    Employ Eq 23 to update the current position of the whale

16:   **end if**

17:  **end for**

18:  Perform Levy flight on each whale using Eq 25

19:  Apply boundary constraints to each search agent and calculate their fitness

20:  Update the optimal whale X*

21:  *t* ← *t* + 1

22: **end while**

23: **return** optimal whale X*

### 4.5 The whole model of EAWOA-KELM

**Fig 4:**
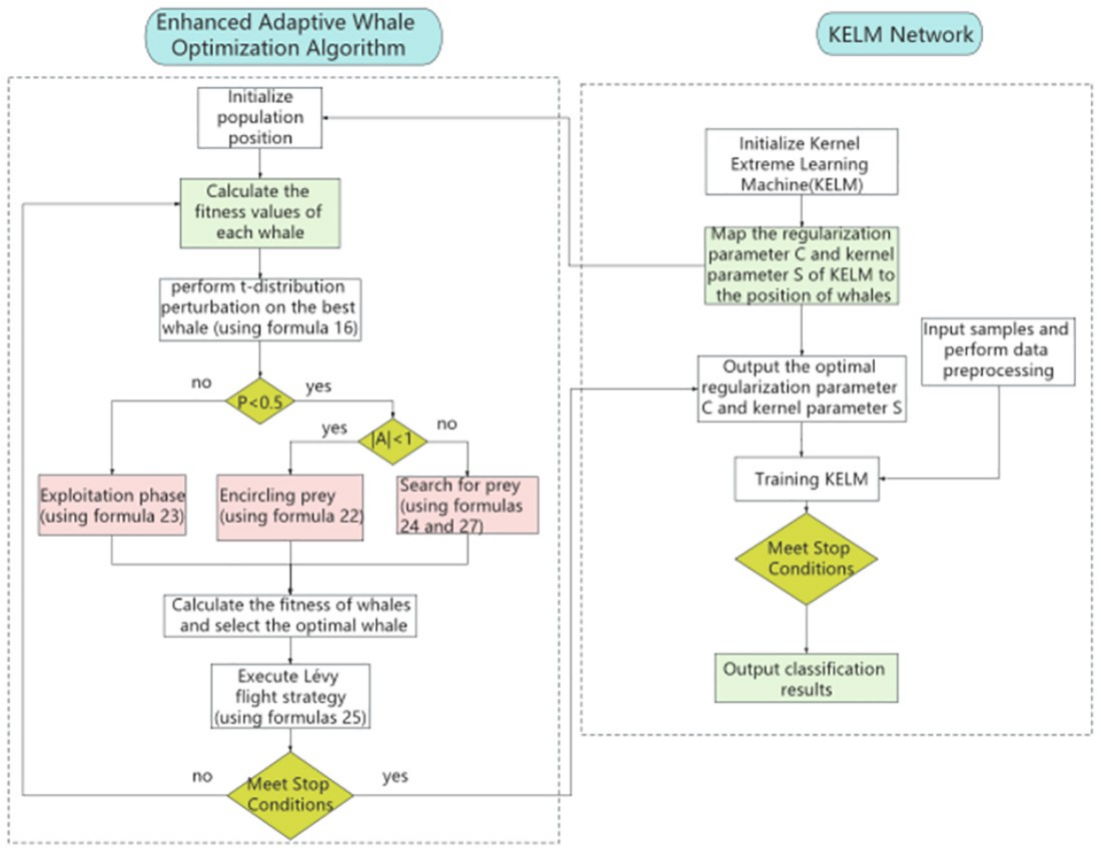
Flowchart of KELM parameters optimized by EAWOA.

Different from other neural network algorithms, the weights of the KELM algorithm are randomly generated, so there is no need to go through the back propagation algorithm. Because of this, the operational efficiency of KELM is very high. However, the manual selection of parameters results in low accuracy of KELM. Therefore, this paper uses the EAWOA algorithm instead of manual parameter selection to increase the precision of KELM. The flowchart of EAWOA optimizing KELM is shown in Fig 4.

Firstly, map the regularization parameter *C* and kernel parameter *S* to the position of whales (*C*, *S*). Additionally, the performance of KELM is related to the fitness function for WOA, which identifying the best combination of whale positions (*C*, *S*) to result in the most precise KELM classification. The entire process divides the data into training and testing sets in a certain proportion, and finally tests the data and outputs the accuracy of classification after the training is completed.

By optimizing the parameters of the KELM algorithm, including kernel functions and regularization parameters, the optimal prediction model can be obtained faster.

## 5 Experiment and result analysis

The experimental part is divided into two parts. In Section 5.1, we assess the effectiveness of the proposed EAWOA. And Section 5.2 proves that the KELM model optimized by EAWOA can be used to improve the accuracy of classification tasks.

### 5.1 Experiment about EAWOA

#### 5.1.1 Algorithm parameter settings and test functions

**Fig 5:**
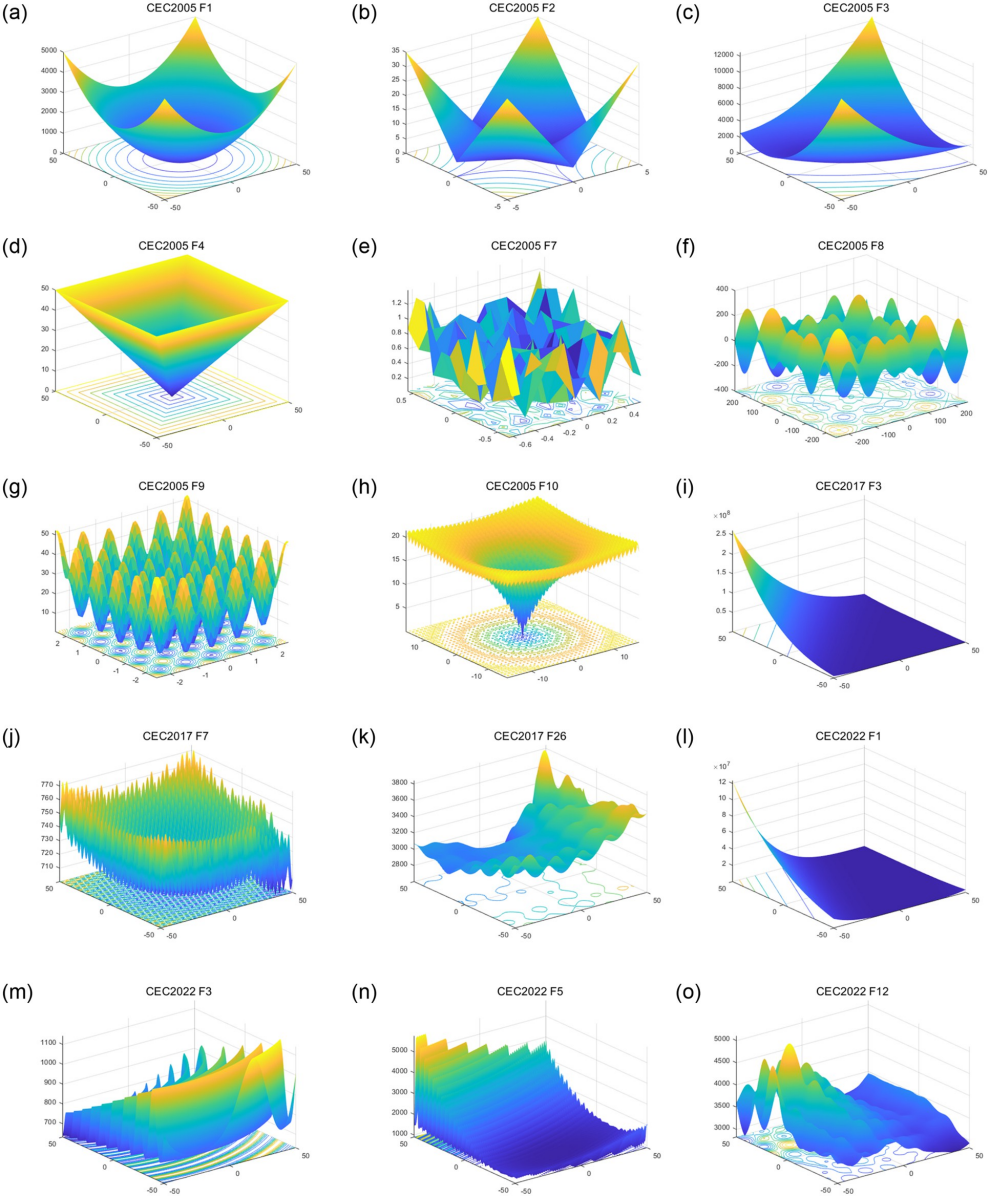
Images of partial benchmark functions.

This paper applied 21 benchmark functions from CEC 2005 [48], CEC 2017 [49] and CEC 2022 [50] to to assess the optimization capabilities of EAWOA. The descriptions of these functions are described in Tables 1–3. The images of some functions are displayed in Fig 5, including both unimodal and multimodal functions. These functions directly reflects optimization problems ranging from simple to complex scenarios. In order to guarantee a just and objective comparison, the parameters have been set as follows: the total population of whales and the count of iterations are set as 100 and 1000 respectively, and dimension parameter settings of each benchmark function are also shown in Tables 1, 2 and 3. All models are run independently 30 times and calculated to obtain two evaluation indicators: mean and standard deviation.

**Table 1:** Descriptions of the selected CEC 2005 benchmark functions.

No	Equation	Search Space	Optimal Value	Dimension
1	$f\left(x\right)=\sum_{i=1}^{n}x_{i}^{2}$	[−100, 100]	0	30
2	$f\left(x\right)=\sum_{i=1}^{n}|x_{i}|+\prod_{i=1}^{n}\left|x_{i}\right|$	[−10, 10]	0	30
3	$f\left(x\right)=\sum_{i=1}^{n}{\left(\sum_{j=1}^{i}x_{j}\right)}^{2}$	[−100, 100]	0	30
4	*f*(**x**) = max_*i*_(|*x*_*i*_|, 1≤i≤n)	[−10, 10]	0	30
5	$f\left(x\right)=\sum_{i=1}^{n}\left[100{\left(x_{i+1}-x_{i}^{2}\right)}^{2}+{\left(x_{i}-1\right)}^{2}\right]$	[−30, 30]	0	30
6	$f\left(x\right)=\sum_{i=1}^{n}ix_{i}^{4}+\text{random}\left[0,1\right)$	[−1.28, 1.28]	0	30
7	$f\left(x\right)=\sum_{i=1}^{n}-x_{i}\text{sin}\left(\sqrt{|x_{i}|}\right)$	[−500, 500]^30^	−418.9829 × 30	30
8	$f\left(x\right)=\sum_{i=1}^{n}\left[x_{i}^{2}-10\text{cos}\left(2\pi x_{i}\right)+10\right]$	[−5.12, 5.12]	0	30
9	$f\left(x\right)=-20\text{exp}(-0.2\sqrt{\frac{1}{n}\sum_{i=1}^{n}x_{i}^{2}})-\text{exp}(\frac{1}{n}\sum_{i=1}^{n}\text{cos}\left(2\pi x_{i}\right))+20+e$	[−32, 32]	0	30
10	$f\left(x\right)=\sum_{i=1}^{n}x_{i}^{2}/4000-\prod_{i=1}^{n}\text{cos}\left(x_{i}/\sqrt{i}\right)+1$	[−600, 600]	0	30

**Table 2:** Descriptions of the selected CEC 2017 benchmark functions.

No	Name	$F_{i}^{*}=F(X_{i}^{*})$	Dimension
11	Shifted and Rotated Rosenbrock’s function	300	100
12	Shifted and Rotated Non-Continuous Rastrigin’ s function	700	30
13	HF 5 (n = 4)	1400	30
14	CF 1 (n = 3)	2000	30
15	CF 7 (n = 6)	2600	30
16	CF 11 (n = 3)	3000	10

**Table 3:** Descriptions of the selected CEC 2022 benchmark functions.

No	Functions	$F_{i}^{*}$	Dimension
17	Shifted and full Rotated Zakharov Function	300	10
18	Shifted and full Rotated Expanded Schaffer’s f6 Function	600	10
19	Shifted and full Rotated Levy Function	900	10
20	Hybrid Function 2 (N = 6)	2000	10
21	Composition Function 4 (N = 6)	2700	10

#### 5.1.2 Comparison of EAWOA with other metaheuristic algorithms

In order to demonstrate the advantages of the put forward model in this paper, it underwent comparison with three popular metaheuristic algorithms: PSO, GWO, and especially the standard WOA. Running independently 30 times under the parameters specified in Section 5.1.1, each algorithm has its average value and standard deviation documented. Improved optimization and algorithm stability are achieved with lower average value and standard deviation. Table 4 shows the statistic results of each model. According to Table 4, the proposed EAWOA demonstrates the best outcomes than the state-of-the-art methods. Especially on the 10 benchmark functions of CEC 2005, it even achieved optimal values on 7 functions, this indicating its competitive in contrast to other exceptional metaheuristic algorithms. In addition, in the 21 benchmark functions of CEC2005, CEC 2017 and CEC 2022, EAWOA achieved better results than the standard WOA, which fully improves its effectiveness and adaptability. These functions include unimodal, multi-front, high-dimensional characteristics, indicating that in complex problems, the EAWOA algorithm can still converge to a higher accuracy, and can even solve for many functions to the theoretical optimal value.

**Table 4:** Statistic results between the proposed algorithm and other metaheuristic algorithms.

Function Number	Metric	PSO	GWO	WOA	EAWOA
1	Mean	6.1986*e* − 02	3.4999*e* − 02	4.0185*e* − 193	0
	Std	3.4999*e* − 02	1.3369*e* − 84	0	0
2	Mean	3.9141*e* − 01	3.0380*e* − 49	7.9521*e* − 113	0
	Std	1.5925*e* − 01	3.8427*e* − 49	4.3214*e* − 112	0
3	Mean	3.6199*e* + 01	2.2587*e* − 27	4.4690*e* + 03	0
	Std	8.9225*e* + 00	7.5058*e* − 27	3.6424*e* + 03	0
4	Mean	1.1606*e* + 00	1.2704*e* − 21	1.4806*e* + 01	0
	Std	1.9810*e* − 01	1.6655*e* − 21	2.0538*e* + 01	0
5	Mean	1.8979*e* + 02	2.6286*e* + 01	2.5891*e* + 01	2.4062*e* + 01
	Std	1.1873*e* + 02	6.1531*e* − 01	2.7858*e* − 01	6.3982*e* + 00
6	Mean	8.3451*e* − 01	2.6507*e* − 04	4.4808*e* − 04	2.1173*e* − 05
	Std	4.6021*e* − 01	1.6394*e* − 04	4.5870*e* − 04	1.5999*e* − 05
7	Mean	−6.8259*e* + 03	−6.4785*e* + 03	−1.1822*e* + 04	−1.2569*e* + 04
	Std	6.5226*e* + 02	7.5203*e* + 02	9.7790*e* + 02	3.0192*e* − 01
8	Mean	8.2294*e* + 01	3.3184*e* − 02	1.8948*e* − 15	0
	Std	2.2991*e* + 01	1.8176*e* − 01	1.0378*e* − 14	0
9	Mean	3.3935*e* − 01	1.0865*e* − 14	3.2863*e* − 15	0
	Std	2.0636*e* − 01	3.2230*e* − 15	2.5380*e* − 15	4.4409*e* − 16
10	Mean	1.8912*e* − 02	0	2.2639*e* − 03	0
	Std	1.5870*e* − 02	0	9.6584*e* − 03	0
11	Mean	3.2144*e* + 05	2.7335*e* + 05	8.1023*e* + 05	3.0270*e* + 05
	Std	5.2820*e* + 04	2.8107*e* + 04	1.3199*e* + 05	1.1894*e* + 04
12	Mean	8.2744*e* + 02	8.4925*e* + 02	1.2308*e* + 03	1.1650*e* + 03
	Std	2.5775*e* + 01	4.0198*e* + 01	7.9554*e* + 01	5.6842*e* + 01
13	Mean	1.7593*e* + 04	3.1515*e* + 05	1.4822*e* + 06	5.9300*e* + 05
	Std	1.7373*e* + 04	4.0024*e* + 05	1.6901*e* + 06	4.7941*e* + 05
14	Mean	2.5850*e* + 03	2.3880*e* + 03	2.8444*e* + 03	2.7326*e* + 03
	Std	1.4889*e* + 02	1.5355*e* + 02	2.0650*e* + 02	1.3724*e* + 02
15	Mean	5.7343*e* + 03	4.4734*e* + 03	7.6356*e* + 03	7.0996*e* + 03
	Std	1.8415*e* + 03	2.8447*e* + 02	1.3030*e* + 03	6.5309*e* + 02
16	Mean	1.9009*e* + 04	6.1800*e* + 05	8.9700*e* + 05	1.6257*e* + 05
	Std	1.4957*e* + 04	9.4334*e* + 05	1.0273*e* + 06	1.7538*e* + 05
17	Mean	3.0000*e* + 02	6.6650*e* + 02	7.7121*e* + 03	4.2001*e* + 03
	Std	4.4405*e* − 05	8.3960*e* + 02	1.1166*e* + 03	4.7782*e* + 02
18	Mean	6.0287*e* + 02	6.0059*e* + 02	6.2725*e* + 02	1.2817*e* + 01
	Std	3.3578*e* + 00	7.8450*e* − 01	6.1988*e* + 02	4.9018*e* + 00
19	Mean	9.0001*e* + 02	9.0115*e* + 02	1.3321*e* + 03	3.1302*e* + 02
	Std	2.7315*e* − 02	7.3501*e* − 01	9.6816*e* + 02	3.7328*e* + 01
20	Mean	2.0223*e* + 03	2.0239*e* + 03	2.0590*e* + 03	2.0550*e* + 03
	Std	1.2229*e* + 01	1.0080*e* + 01	1.6452*e* + 01	8.6518*e* + 00
21	Mean	2.8674*e* + 03	2.8639*e* + 03	2.8806*e* + 03	2.8685*e* + 03
	Std	2.5913*e* + 01	2.0693*e* + 00	2.2083*e* + 01	1.1350*e* + 00

Simultaneously, the analysis reveals that the standard deviation of EAWOA is consistently lower than that of WOA, with the exception of the F5 test function discussed in this study. In comparison to all algorithms, EAWOA has attained the best standard deviation value on the majority of test functions, especially in the seven functions such as F1, F2, and F3, the standard deviation mean has reached the ideal value of 0. The experimental findings indicate that the EAWOA algorithm exhibits higher convergence stability and better robustness compared to other algorithms such as PSO, GWO, and WOA.

#### 5.1.3 Comparison of EAWOA with other WOA variants

In order to reflect on the advantages of the EAWOA, this part selects WOA and 4 other rencent WOA algorithm variants for comparison: WOA_LFDE [41], eWOA [51], MWOA [52] and MSWOA [53]. Likewise, 30 independent trials were performed on EAWOA and other upgraded WOA, employing the identical parameters as described in Section 5.1.1, and the final averages and standard deviations were calculated.

Table 5 presents the comparison results, indicating that EAWOA exhibits favorable performance: With a record of 10 wins, 10 losses, and 1 draw, it ranks the same as WOA_LFDE. Compared to the other three improved models, it has obvious advantages. Compared to EWOA, it won on 9 benchmark functions, lost on 5 benchmark functions, and tied on 7 benchmark functions. Compared to MWOA, it won on 13 benchmark functions, lost on 1 test function, and benchmark on 7 test functions. Compared to MSWOA, it won on 16 benchmark functions, lost on 2 benchmark functions, and tied on 3 benchmark functions. The results above demonstrate that EAWOA outperforms individual improved WOA algorithms more frequently on test functions, and it attains the theoretical optimal values on seven functions, such as F1, F2, and F3. When it comes to standard deviation, EAWOA consistently shows smaller values compared to other advanced algorithms on 14 out of 21 functions, highlighting its stability advantage.

**Table 5:** Statistical results of various algorithms.

Function number	Metric	WOA	eWOA	WOA_LFDE	MWOA	MSWOA	EAWOA
1	Mean	2.2451*e* − 193	0	8.2185*e* − 91	0	1.0286*e* − 310	0
	Std	0	0	2.1890*e* − 90	0	0	0
2	Mean	2.2384*e* − 112	0	2.6943*e* − 59	0	2.1544*e* − 166	0
	Std	1.2233*e* − 111	0	1.0714*e* − 58	0	0	0
3	Mean	5.1483*e* + 03	0	1.1235*e* − 01	0	1.9849*e* − 276	0
	Std	3.4690*e* + 03	0	1.8444*e* − 01	0	0	0
4	Mean	1.8278*e* + 01	0	8.8411*e* + 00	0	1.8847*e* − 139	0
	Std	2.5145*e* + 01	0	7.0723*e* + 00	0	1.9239*e* − 139	0
5	Mean	2.5925*e* + 01	2.6208*e* + 01	1.6862*e* + 01	2.8213*e* + 01	7.2200*e* + 00	2.2344*e* + 01
	Std	1.6978*e* − 01	3.8235*e* − 01	1.6957*e* + 00	4.0623*e* − 01	1.2167*e* + 01	8.6945*e* + 00
6	Mean	6.7497*e* − 04	1.6798*e* − 05	4.9273*e* − 03	1.1644*e* − 05	2.6835*e* − 05	2.1226*e* − 05
	Std	1.0009*e* − 03	2.0007*e* − 05	2.4575*e* − 03	1.1486*e* − 05	2.6104*e* − 05	1.5880*e* − 05
7	Mean	−1.2033*e* + 04	−1.2368*e* + 04	−7.3266*e* + 03	−6.1861*e* + 03	−1.0120*e* + 04	−1.2569*e* + 04
	Std	6.6854*e* + 02	4.2508*e* + 02	4.6229*e* + 02	1.5205*e* + 03	1.9560*e* + 03	2.4307*e* − 01
8	Mean	5.6843*e* − 15	0	4.5072*e* + 01	0	0	0
	Std	2.2884*e* − 14	0	1.9893*e* + 01	0	0	0
9	Mean	3.5231*e* − 15	4.4409*e* − 16	7.7228*e* − 02	4.4409*e* − 16	4.4409*e* − 16	4.4409*e* − 16
	Std	2.9108*e* − 15	0	4.2300*e* − 01	0	0	0
10	Mean	8.7486*e* − 04	0	5.6624*e* − 03	0	0	0
	Std	4.7918*e* − 03	0	7.9725*e* − 03	0	0	0
11	Mean	7.9794*e* + 05	2.9539*e* + 05	3.4782*e* + 05	3.5482*e* + 05	3.6472*e* + 05	2.9740*e* + 05
	Std	1.5755*e* + 05	1.6648*e* + 04	9.9086*e* + 04	6.2023*e* + 03	6.9677*e* + 03	1.3552*e* + 04
12	Mean	1.2102*e* + 03	1.2281*e* + 03	1.1533*e* + 03	1.4280*e* + 03	1.3426*e* + 03	1.1537*e* + 03
	Std	9.6573*e* + 01	6.1251*e* + 01	1.3183*e* + 02	3.9180*e* + 01	7.0033*e* + 01	3.6128*e* + 01
13	Mean	1.0255*e* + 06	1.3330*e* + 05	2.0903*e* + 03	8.6163*e* + 06	1.7048*e* + 06	6.8324*e* + 05
	Std	1.1032*e* + 06	1.8877*e* + 05	9.4018*e* + 02	4.9885*e* + 06	1.5616*e* + 06	4.0137*e* + 05
14	Mean	2.7864*e* + 03	2.6739*e* + 03	2.7392*e* + 03	3.0443*e* + 03	2.8024*e* + 03	2.6722*e* + 03
	Std	1.8757*e* + 02	1.7574*e* + 02	2.3670*e* + 02	1.8942*e* + 02	1.5186*e* + 02	1.7649*e* + 02
15	Mean	7.3953*e* + 03	8.1697*e* + 03	6.4169*e* + 03	1.1200*e* + 04	7.8806*e* + 03	7.3936*e* + 03
	Std	1.1830*e* + 03	1.3157*e* + 03	1.4413*e* + 03	7.0865*e* + 02	9.3991*e* + 02	6.5663*e* + 02
16	Mean	4.4635*e* + 05	2.2827*e* + 06	2.5183*e* + 05	4.9144*e* + 06	6.3159*e* + 04	2.7027*e* + 05
	Std	4.3690*e* + 05	2.3627*e* + 06	4.6924*e* + 05	5.4889*e* + 06	7.9347*e* + 04	4.7504*e* + 05
17	Mean	9.0215*e* + 03	2.0420*e* + 03	3.0000*e* + 02	6.2976*e* + 03	5.6947*e* + 03	1.1591*e* + 03
	Std	4.0021*e* + 03	1.3253*e* + 03	3.8417*e* − 11	1.6050*e* + 03	2.1965*e* + 03	4.8063*e* + 02
18	Mean	6.3012*e* + 02	6.2754*e* + 02	6.1449*e* + 02	6.4730*e* + 02	6.2649*e* + 02	6.1965*e* + 02
	Std	1.4875*e* + 01	7.5734*e* + 00	8.7851*e* + 00	8.4407*e* + 00	1.3869*e* + 01	4.9728*e* + 00
19	Mean	1.2700*e* + 03	1.1672*e* + 03	1.1500*e* + 03	1.4754*e* + 03	1.3137*e* + 03	9.9104*e* + 02
	Std	1.7587*e* + 02	1.0712*e* + 02	1.6843*e* + 02	1.4960*e* + 02	2.2006*e* + 02	6.5112*e* + 01
20	Mean	2.0597*e* + 03	2.0548*e* + 03	2.0379*e* + 03	2.1063*e* + 03	2.0714*e* + 03	2.0551*e* + 03
	Std	1.8362*e* + 01	1.6064*e* + 01	2.5340*e* + 01	2.2666*e* + 01	3.9723*e* + 01	1.0382*e* + 01
21	Mean	2.8822*e* + 03	2.8808*e* + 03	2.8672*e* + 03	3.1007*e* + 03	2.8797*e* + 03	2.8680*e* + 03
	Std	1.9984*e* + 01	1.8692*e* + 01	4.5681*e* + 00	7.9312*e* + 01	3.3627*e* + 00	1.3332*e* + 00

In this experiment, it is apparent that the EAWOA algorithm not only surpasses the standard WOA in the context of global and local search capabilities, but also exhibits considerable benefits over other improved versions of WOA.

#### 5.1.4 Convergence speed analysis

**Fig 6:**
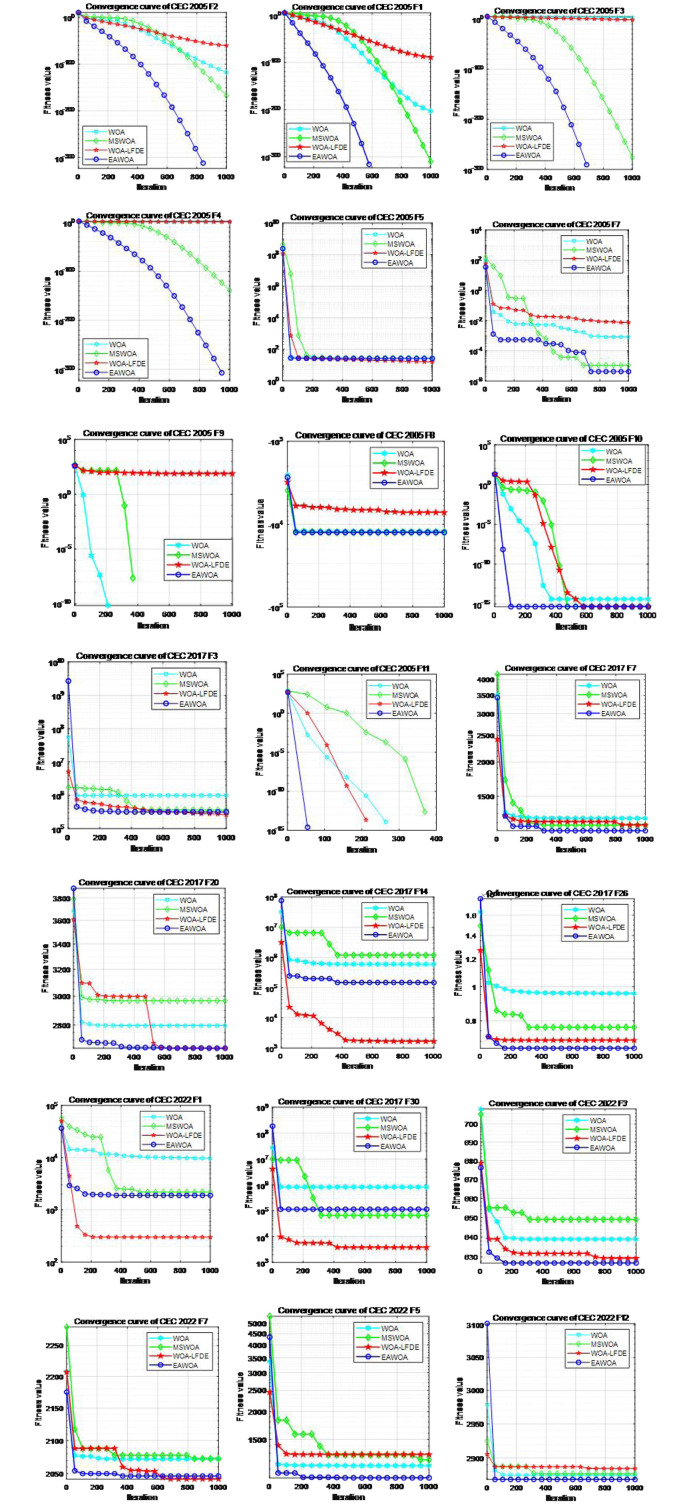
Convergence curves of various improved WOA algorithms.

In addition to assessing the fitness value, the EAWOA algorithm’s efficiency can also be evaluated by convergence speed. This section delves into the benefits of its fast convergence speed. Fig 6 offers a comparison between EAWOA and standard WOA, the improved MSWOA and WOA_LFDE. The nonlinear convergence parameters *α* and *ω* can skilfully strengthen convergence. Simultaneously, other strategies also perform a key function in finding the global optimum, helping EAWOA quickly approach the optimal value. In contrast to the other three algorithm, the convergence curve of the EAWOA algorithm decreases rapidly, making it possible to quickly reach the theoretical optimal solution for most functions. The trend in Fig 6 shows a significant decrease in the evolution curve of the EAWOA algorithm as the number of iterations increases. It has the ability to rapidly reach the best solution when dealing with numerous basic test functions like F1-F4, including single-mode functions. Even reaching convergence on many functions hundreds of iterations earlier than other algorithms, such as F11 of CEC 2005. Furthermore, EAWOA shows considerable advantages in convergence speed for other multi-modal or mixed functions. By comparing with Table 5, it is evident that EAWOA not only exhibits faster convergence speed but also showcases high optimization accuracy, demonstrating fast convergence and strong global search capabilities. Although WOA_LFDE obtained the same raking in the previous experiments as EAWOA, it exhibited slower convergence compared to the EAWOA algorithm.

The experiments in Sections 5.1.1 to 5.1.4 prove that the EAWOA model proposed in this paper has considerable advantages compared with some current metaheuristic algorithms, standard WOA, and some excellent variants of WOA. In addition, EAWOA can also reach convergence faster during the iterative process.

### 5.2 Experiment about EAWOA-KELM

#### 5.2.1 Classification datasets

This paper uses seven five classification datasets to validate the advantages of the improved KELM: SDAS [54], NPHA [55], TME [56], SRP [57], Abalone [58], Balance-Scale [59] and Glass [60]. SDAS is a dataset which related to students’ performance in high education. It contains 36 features and 3 classifications. 36 features include gender, academic performance, parental qualification, etc., while classifications include dropout, enrolled, and graduate. In order to ensure even distribution of data, we took 500 samples for each category of students, totaling 1500 samples. NPHA is a dataset that predicts the number of doctors an elderly person visits within a year. It has 714 examples, including 14 features and 3 categories. TME is a dataset that classifies music based on emotions. It contains 400 examples and uses 50 features to divide music into 4 emotional types: happy, sad, angry, and relax. SPR is a dataset for accent detection and recognition, it contains 329 individual English words, spoken by native speakers originating from six different nations. And the final Abalone is a dataset predicts the age of abalone based on 8 characteristics. In order to make the classification dataset more uniform, we selected 100 samples for each category from 5 to 14 years old, forming a total of 1000 samples. The dataset Balance-Scale illustrates the balance results of a scale, consisting of 625 data points, each with 4 attributes and a category indicating whether the scale is tilted to the left, tilted to the right, or in balance. The last data set Glass consists of 214 data points and utilizes 9 attributes to categorize 6 different types of glass.

#### 5.2.2 EAWOA-KELM for classification data tasks

In this section, 7 models related to KELM are used to classify the 5 datasets mentioned above, namely namely KELM, SSA-KELM, GTO-KELM, eWOA-KELM, WOA_LFDE-KELM, MWOA-KELM, MSWOA-KELM and our proposed EAWOA-KELM. Following the dataset introduced in Section 5.2.1, the experimental parameters are set as follows: After randomizing the data set in each classification task, split it into a training set and a test set using a 4:1 ratio. The population size for enhancing KELM through metaheuristic algorithm is set to 40, and the dimension to 2, which represents the two optimized parameters C and S, the iterations number is set to 100. Evaluation was based on accuracy, recall, and F1 score, with the average value computed over five runs. The outcomes are detailed in Table 6.

**Table 6:** Performance metrics of various classifiers on different datasets.

Dataset	Classifier	Accuracy (%)	Recall (%)	F1 Score (%)
SDAS [54]	KELM	79.000	79.000	78.966
	SSA-KELM	81.500	81.500	81.496
	GTO-KELM	79.500	79.500	79.458
	WOA-KELM	80.000	80.000	79.982
	eWOA-KELM	82.000	82.000	81.984
	WOA_LFDE-KELM	77.000	77.000	76.812
	MWOA-KELM	81.500	81.500	81.488
	MSWOA-KELM	80.000	80.000	79.968
	EAWOA-KELM	83.500	83.500	83.500
NPHA [55]	KELM	47.183	32.583	28.145
	SSA-KELM	53.521	37.323	32.559
	GTO-KELM	52.817	39.077	36.904
	WOA-KELM	50.000	34.728	30.004
	eWOA-KELM	52.817	37.361	33.693
	WOA_LFDE-KELM	51.408	34.256	27.458
	MWOA-KELM	52.113	35.393	29.630
	MSWOA-KELM	53.521	36.293	30.346
	EAWOA-KELM	54.225	36.401	29.744
TME [56]	KELM	85.000	85.000	84.896
	SSA-KELM	93.333	93.333	93.284
	GTO-KELM	91.667	91.667	91.664
	WOA-KELM	88.333	88.333	88.198
	eWOA-KELM	91.667	91.667	91.697
	WOA_LFDE-KELM	90.000	90.000	90.037
	MWOA-KELM	88.333	88.333	88.188
	MSWOA-KELM	90.000	90.000	90.191
	EAWOA-KELM	95.000	95.000	94.994
SPR [57]	KELM	81.667	63.333	68.770
	SSA-KELM	88.333	79.394	80.602
	GTO-KELM	86.667	78.788	81.971
	WOA-KELM	85.000	77.677	81.997
	eWOA-KELM	88.333	81.616	84.429
	WOA_LFDE-KELM	86.667	77.172	78.380
	MWOA-KELM	85.000	75.455	77.061
	MSWOA-KELM	83.333	69.394	73.614
	EAWOA-KELM	90.000	83.838	86.768
Abalone [58]	KELM	38.333	38.333	39.130
	SSA-KELM	47.000	47.000	47.385
	GTO-KELM	44.167	44.167	42.662
	WOA-KELM	45.000	45.000	44.274
	eWOA-KELM	51.667	51.667	50.735
	WOA_LFDE-KELM	47.500	47.500	47.251
	MWOA-KELM	49.167	49.167	47.061
	MSWOA-KELM	46.667	46.667	44.785
	EAWOA-KELM	50.833	50.833	50.272
Balance-Scale [59]	KELM	24.000	24.000	19.043
	SSA-KELM	28.800	28.800	23.452
	GTO-KELM	32.000	32.000	25.257
	WOA-KELM	31.200	31.200	20.508
	eWOA-KELM	27.200	27.200	18.321
	WOA_LFDE-KELM	30.400	30.400	19.633
	MWOA-KELM	31.200	31.200	20.510
	MSWOA-KELM	30.400	30.400	21.905
	EAWOA-KELM	32.000	32.000	18.767
Glass [60]	KELM	86.486	68.667	69.613
	SSA-KELM	91.892	76.667	80.083
	GTO-KELM	94.595	83.333	85.381
	WOA-KELM	94.595	86.667	88.750
	eWOA-KELM	89.189	78.667	73.500
	WOA_LFDE-KELM	91.892	81.905	84.676
	MWOA-KELM	94.595	86.667	90.750
	MSWOA-KELM	91.892	90.571	90.467
	EAWOA-KELM	97.297	90.000	90.476

From Table 6, it can be seen that the classification results of KELM on the dataset have significantly improved after being optimized by different improved models. This indicates that the use of metaheuristic algorithms to optimize (C, S) parameter combinations has a significant effect on the improvement of KELM models. In the evaluation involving seven datasets, it was found that EAWOA-KELM outperformed WOA-KELM in classification tasks, showing an improvement of more than 5% in certain datasets among the nine models tested. In comparison to other enhanced algorithms, EAWOA-KELM outperformed them, showcasing the strong global search capability of the EAWOA algorithm in this study and its tendency to avoid getting stuck in local optima while tuning KELM parameters.

#### 5.2.3 Experimental selection of parameters *β*_*min*_ and *β*_*max*_

This paper introduces a new inertia weight *ω* to the position update of the enhanced whale optimization algorithm, as outlined in formulas 22-24, with the expression of provided in formulas 20 and 21. Altering the values of and in will lead to diverse optimization results with the EAWOA algorithm.

In order to quickly determine the optimal value range of parameters and, this paper selects the SDAS data set in Table 6 as the standard data set for testing. The EAWOA-KELM model algorithm was executed 5 times, with the average values of accuracy, recall, and F1 score serving as the basis for result assessment. The detailed results are provided in Table 7.

**Table 7:** Performance metrics for different *β*_min_ and *β*_max_ values.

*β*_min_	*β*_max_	Accuracy (%)	Recall (%)	F1 Score (%)	*β*_min_	*β*_max_	Accuracy (%)	Recall (%)	F1 Score (%)
0.1	0.9	82.000	82.000	82.000	0.1	0.7	83.000	83.000	83.000
0.2	0.9	80.000	80.000	79.837	0.2	0.7	83.000	83.000	82.985
0.3	0.9	77.000	77.000	76.917	0.3	0.7	80.000	80.000	79.998
0.4	0.9	83.500	83.500	83.500	0.4	0.7	81.500	81.500	81.488
0.5	0.9	77.500	77.500	77.432	0.5	0.7	83.000	83.000	82.993
0.1	0.8	77.500	77.500	77.495	0.1	0.6	83.500	83.500	83.480
0.2	0.8	80.000	80.000	79.950	0.2	0.6	80.500	80.500	80.441
0.3	0.8	81.500	81.500	81.444	0.3	0.6	79.500	79.500	79.495
0.4	0.8	79.500	79.500	79.438	0.4	0.6	81.000	81.000	80.992
0.5	0.8	81.000	81.000	80.998	0.5	0.6	79.000	79.000	78.992

*β*_*min*_ and *β*_*max*_ values set the highest and lowest boundaries for the extent of individual position adjustment in the EAWOA algorithm, specifically defining the level of self-preservation. Hence, this parameter plays a crucial role in determining the ultimate classification outcome. To discover a more effective pairing of *β*_*min*_ and *β*_*max*_ within a practical range, this study employs a binary strategy. Within the *β*_*max*_ range of 0.6 to 0.9 and the *β*_*min*_ range of 0.1 to 0.5, it has been established that 20 combinations encompass intervals varying between 0 and 1 in size. The impact of *β*_*min*_ and *β*_*max*_ values on the results is evident from the findings in Table 7. Variances in these values lead to differences of more than 6% in accuracy, recall, and F1 score. Experiments show that when *β*_*max*_ is fixed, the best result is achieved when *β*_*min*_ is 0.5 less than *β*_*max*_. Within the group, (0.4, 0.9), (0.3, 0.8), and (0.1, 0.6) all excelled in the three indicators. (0.2, 0.7) outperformed the rest in both indicators and is just 0.008% lower than (0.1, 0.7) in F1 score. The most favorable outcomes were achieved by (0.4, 0.9) among these combinations.

## 6 Conclusions and future work

The kernel extreme learning machine holds significant importance in the realm of machine learning and is extensively employed for data classification. To optimize its efficiency for solving classification problems, an innovative model EAWOA, is proposed in this paper. Multiple strategies were used in EAWOA, including innovative T-distribution perturbations, nonlinear parameters, novel Levy flight, and surrounding prey strategy by 3 excellent whales. This improved algorithm has successfully addressed the problems that are inherent in the original WOA. Based on 21 benchmark functions and 7 classification datasets, the experimental results have demonstrated the superiority of EAWOA and EAWOA-KELM models in global search, convergence speed, classification accuracy, and other aspects. Utilizing a fusion of whale algorithm and kernel extreme learning machine, this technique elevates the data classification accuracy and efficiency.

Despite the positive experimental results demonstrating the effectiveness of EAWOA in optimizing KELM, there are still some limitations. The following are the main limitations of EAWOA. First, EAWOA’s main limitations include the need for resetting after each optimization process, despite its ability to potentially optimize parameters for a wider range of machine learning classifiers. Second, the incorporation of extra Levy random flights and adaptive factors in EAWOA increases its complexity, leading to a higher demand for computing resources and longer execution times. Hence, further research is essential to enhance the shortcomings of this model. EAWOA will be employed in upcoming studies to enhance the performance of additional classifiers. The next focus will also explore ways to decrease the computational complexity of the model in order to reduce the overall running time. Moreover, EAWOA-KELM can also make more flexible adjustments to deal with constrained optimization problems.
